# Supplementary material for: Ribosomal 18S rRNA base pairs with mRNA during eukaryotic translation initiation
Source: Nat Commun. 2016 Aug 24;7:12622. doi: 10.1038/ncomms12622 (PMC4999511; doi:10.1038/ncomms12622)
Supplement: Supplementary Information — Supplementary Figures 1-7 [file ncomms12622-s1.pdf]

Supplementary Figure 1

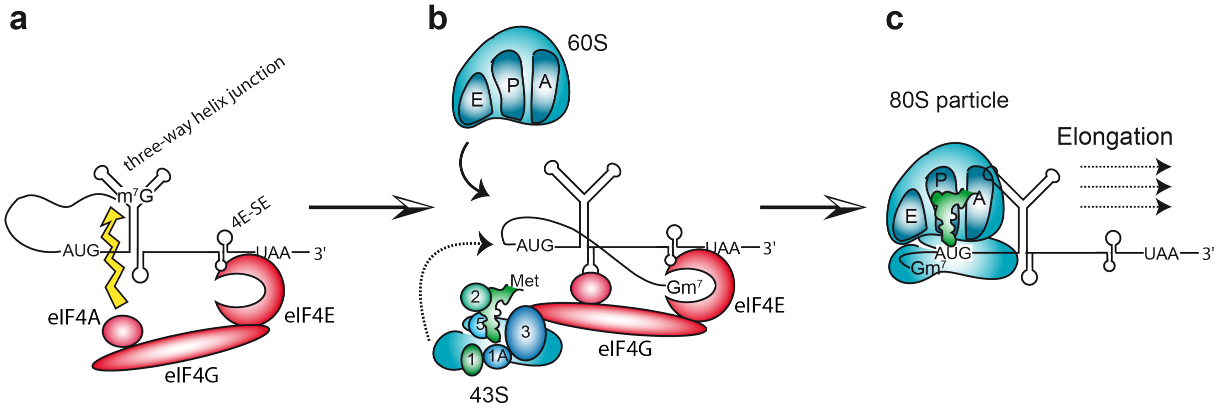

Supplementary Figure 1 | Model for translation initiation of histone H4 mRNA[14]. a-b, eIF4E, as part of eIF4F, interacts *via* its N-terminal moiety with the 4E-SE structure. The amino-terminal peptide from eIF4E plays a crucial role during the binding. The interaction of the cap with the cap-binding pocket located in the three-way helix junction occludes binding to eIF4E. The helicase eIF4A releases the 5'UTR from the three-way helix junction allowing direct binding of the 43S particle onto the AUG initiation codon. **c,** Once the mRNA structure has been unfolded and the 80S ribosome assembled, the elongation process can start.

Supplementary Figure 2

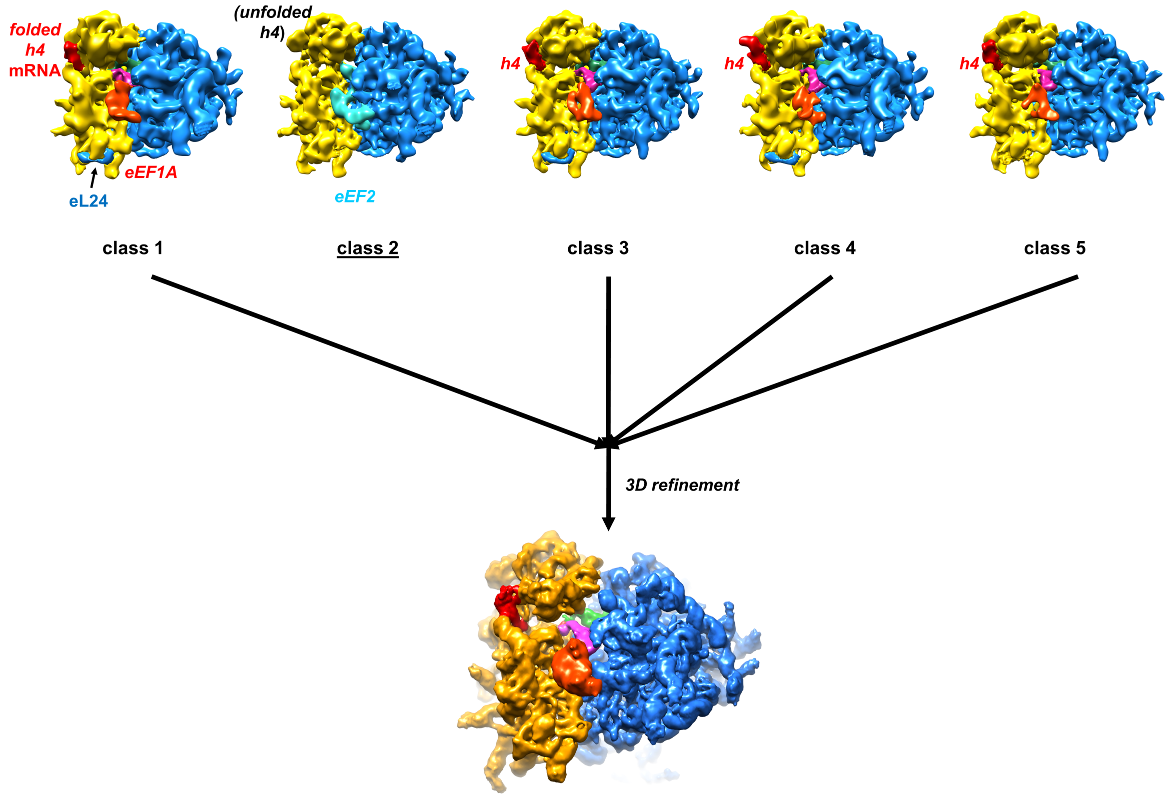

Supplementary Figure 2 | Cryo-EM image processing and 3D classification scheme.

Supplementary Figure 3

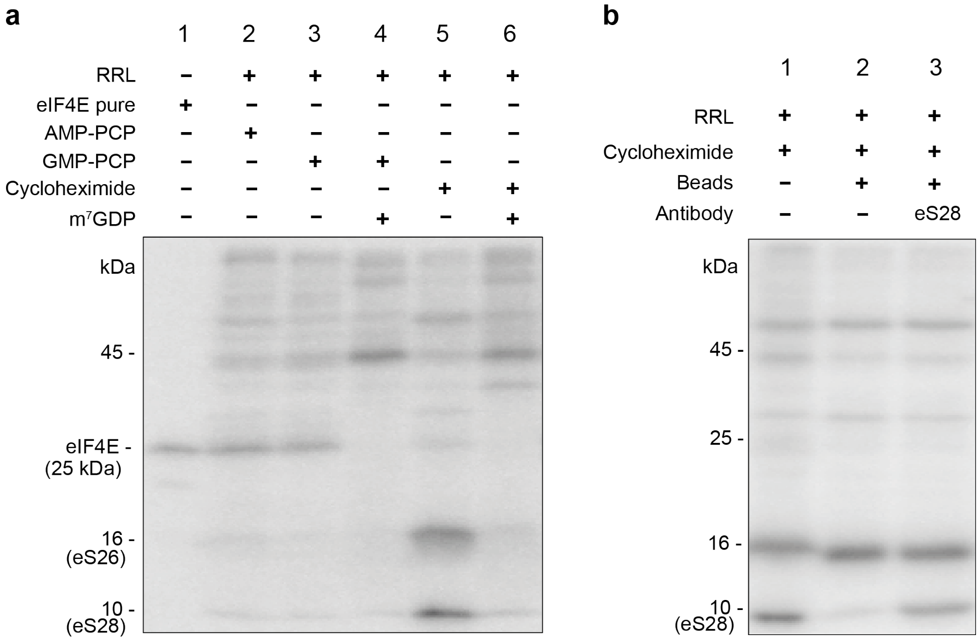

Supplementary Figure 3 | Chemical cross-linking of the 5' cap and immuno-precipitation of ribosomal proteins. a, *h4* mRNA, radiolabelled at the level of the G of the cap, was periodate-oxidized before assembling initiation complexes in reticulocytes extracts in the presence of various inhibitors or cycloheximide (see methods). After RNase digestion, samples were fractionated on SDS-polyacrylamide gel. In the presence of AMP-PCP or GMP-PNP, which blocks ribosome assembly at the 48S step, eIF4E was cross-linked by the 5' cap of *h4* (lanes 1-3). The specificity of the cross-link was confirmed by adding the cap analogue m^7GDP that induced release of the cap from eIF4E and disappearance of the cross-link (lane 4). Cycloheximide induces accumulation of 80S particles by blocking translation at the translocation step. In the presence of cycloheximide, two cross-linked proteins of 10 and 16 kDa were detected (lane 5). The two cross-links disappeared in the presence of cap analogue m^7GDP (lane 6). This suggests the presence of a specific cap-binding pocket on the ribosome. The two cross-linked proteins p10 and p16 are matching with the sizes of eS28 and eS26 that are both located on the exit site of the mRNA cleft on the small ribosomal subunit. **b,** eS28 was further immuno-precipitated with specific antibodies coupled to MagnaBind™ Protein G beads (Thermo Scientific), confirming the identity of the protein. Lane 1 shows the input after cross-linking and RNase A treatment. Lane 2 shows the same sample bound to the beads without antibody binding. The protein corresponding to the 16 kDa band was unfortunately binding aspecifically the beads and could not be formally attributed to eS26. Lane 3 shows the specific immuno-precipitation of eS28. Rabbit antibody against human eS26 was from Novus (NBP1-92353).

Supplementary Figure 4

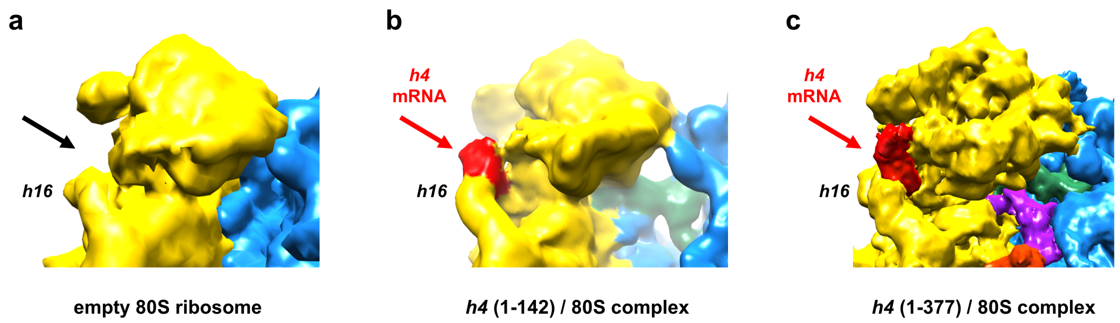

80S ribosome complex	empty 80S	h4 (1-142) / 80S	h4 (1-377) / 80S
Electron microscope	Polara	Polara	Titan Krios
CMOS electron detector	Falcon I	Falcon I	Falcon II
Acceleration voltage [kV]	300	300	300
Magnification	59 000	59 000	59 000
Pixel size [Å]	1.36	1.36	1.1

Supplementary Figure 4 | Comparison of the cryo-EM structures of the empty 80S, a 3'-truncated *h4*/80S complex and the full-length *h4*/80S ribosome complex. Data were collected on two different microscopes with slightly different settings.

Supplementary Figure 5

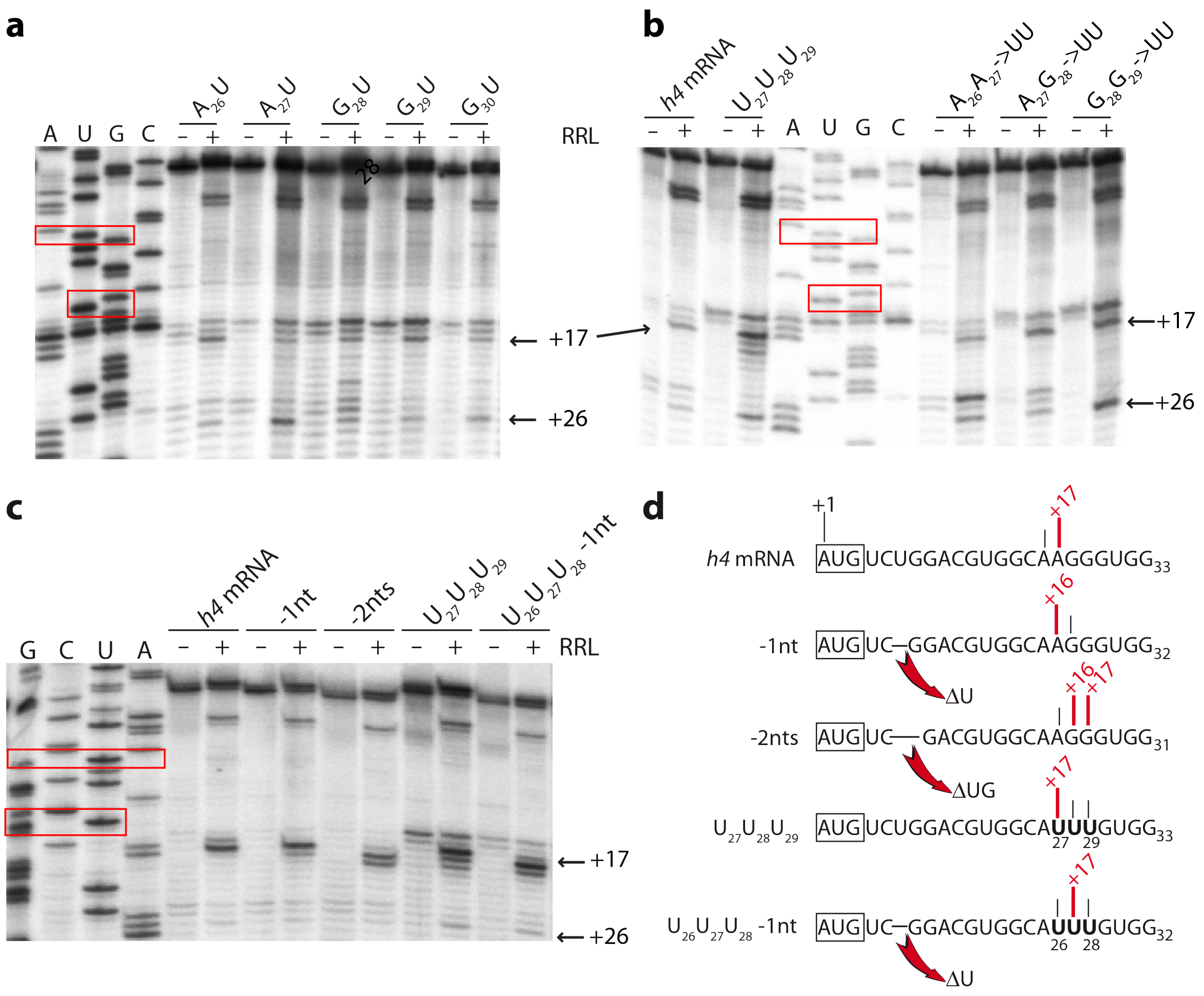

Supplementary Figure 5 | Ribosome toe-prints on different mutants of *h4* mRNA. Initiation complexes were assembled in RRL extracts in the presence of cycloheximide/hygromycin B to stall the initiation complexes on the AUG codon. Reaction samples were separated on 8% denaturing PAGE together with the appropriate dideoxy-nucleotide sequencing ladder. Positions of the toe-prints are indicated, starting from the A of AUG codon. AUG initiation codon and GUG codon are boxed. **a-b,** Toe-prints performed on single and double mutants. AUG initiation codon and GUG codon are boxed in red. **c,** Toe-prints performed on the triple mutant and deletion mutants. Sequence of the synthesized DNA fragments is shown on the right. For clarity, only the sequence of wild-type *h4* is reported on the left. Although some band locations are similar on the gel, their positions relative to the AUG may differ depending to the deletion (see panel d). **d,** Partial sequences of *h4* mRNA and mutated derivatives. Toe-prints from panel c are indicated and numbered starting from the first A of the AUG codon.

Supplementary Figure 6

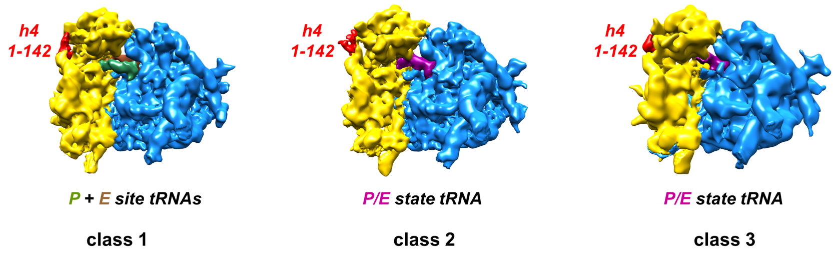

Supplementary Figure 6 | Structure comparison of sub-classes of the 3'-truncated *h4 (1-142)*/80S complex showing different tRNA states and 40S head movements.

Supplementary Figure 7

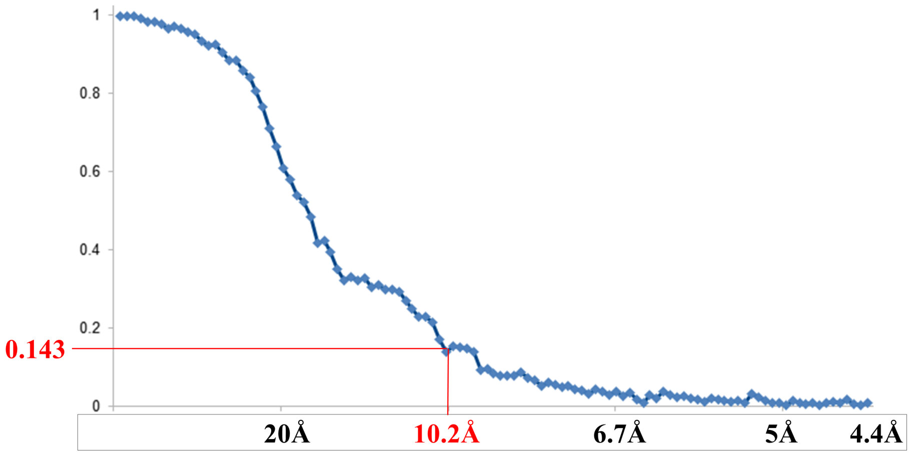

Supplementary Figure 7 | Resolution estimation. The average resolution o the cryo-EM map (*h4*/80S ribosome complex) as estimated from Fourier shell correlation according to the 0.143 criterion (in red; see methods).
